# Role of a 49 kDa *Trypanosoma cruzi* Mucin-Associated Surface Protein (MASP49) during the Infection Process and Identification of a Mammalian Cell Surface Receptor

**DOI:** 10.3390/pathogens12010105

**Published:** 2023-01-07

**Authors:** Bertha Espinoza, Ignacio Martínez, María Luisa Martínez-Velasco, Miriam Rodríguez-Sosa, Augusto González-Canto, Alicia Vázquez-Mendoza, Luis I. Terrazas

**Affiliations:** 1 Departamento de Inmunología, Instituto de Investigaciones Biomédicas, Universidad Nacional Autónoma de México, Ciudad de México C.P. 04510, Mexico; 2 Unidad de Investigación en Biomedicina, Facultad de Estudios Superiores Iztacala, Universidad Nacional Autónoma de México, Tlalnepantla C.P. 54090, Mexico; 3 Departamento de Medicina Experimental, Facultad de Medicina, Universidad Nacional Autónoma de México, Ciudad de México C.P. 04510, Mexico

**Keywords:** *Trypanosoma cruzi*, MASP family, infection process, mMGL

## Abstract

*Trypanosoma cruzi* is the etiologic agent of Chagas disease, a parasitic disease of great medical importance on the American continent. Trypomastigote infection’s initial step in a mammalian host is vital for the parasite’s life cycle. A trypomastigote’s surface presents many molecules, some of which have been proposed to be involved in the infection process, including a glycoprotein family called mucin-associated surface proteins (MASPs). This work describes a 49-kDa molecule (MASP49) that belongs to this family and is expressed mainly on the surfaces of amastigotes and trypomastigotes but can be found in extracts and the membrane-enriched fractions of epimastigotes. This protein is partially GPI-anchored to the surface and has a role during the internalization process, since its blockade with specific antibodies decreases parasite entry into Vero cells by 62%. This work shows that MASP49 binds to peritoneal macrophages and rat cardiomyocytes, undergoes glycosylation via galactose N-acetylgalactosamine, and can attach to the macrophage murine C-type lectin receptor (mMGL). These results suggest that MASP49 can be considered a virulence factor in *T. cruzi*, and a better understanding of its role in the infection process is necessary.

## 1. Introduction

The hemoflagellate parasite *Trypanosoma cruzi* is the causative agent of Chagas disease or American trypanosomiasis. Chagas disease is the most important parasitosis, concerning the number of affected people and related deaths on the American continent [1]. Chagas disease has two main phases: the acute phase and the chronic phase. The acute phase occurs immediately after the parasite enters a host and is characterized by the presence of the parasite in the bloodstream. Symptoms during this phase are nonspecific and include malaise, headache, and fever. The chronic phase occurs following the disappearance of the parasite from the bloodstream and can last many years without clinical manifestations. However, approximately 30% of individuals may develop symptoms and present a so-called symptomatic chronic phase. The primary pathologies developed during this stage are cardiomyopathy and, to a lesser extent, megaviscera (heart, esophagus, and colon) [1].

The metacyclic trypomastigote is the parasite stage that attaches to cells and infects a vertebrate host. Upon entering the organism, it can infect virtually any cell type [2]. The trypomastigote cell surface is covered with many proteins, including trans-sialidase and mucin family members. Some of these proteins have been proposed as ligands for host cells, although accurate data regarding receptor recognition has not been reported. Identifying parasite surface molecules that act as ligands and participate in binding to host cells is a current area of research related to this pathogen.

Although there is some knowledge about surface proteins in *T. cruzi* [3], new data are still emerging. A new family of surface proteins was described following the publication of the complete genome of this parasite in 2005 [4]. Because the genes of this family are located within blocks of genes that belong to the mucin and trans-sialidase families, this new family was named mucin-associated surface proteins (MASPs). A total of 1377 genes were identified, of which almost two-thirds (771) are coding genes; the remaining one-third are pseudogenes. Sequence analysis of this protein family has revealed that its N- and C-terminal regions are highly conserved and contain a signal peptide and a GPI anchorage site, suggesting localization to the cell surface [4]. A study that compared sequences between Sylvio X10/1 (lineage 1) and CL Brener (lineage VI) found fewer MASP genes in the former lineage [5]. Some works have suggested that the similarities and polymorphism found in the members of this family of proteins could allow the identification of up to seven subgroups [6].

The first proteomic data for this family indicated that MASPs might have low expression levels, given that only four proteins were identified in the trypomastigote stage and one in the epimastigote stage. It has been suggested that MASPs are expressed during the intermediate stages of the parasite, or that they may be subjected to post-translational modifications, such as different N-glycosylation [7]. Other reports about these genes showed that proteins are expressed in epimastigotes, trypomastigotes, and amastigotes, and that MASPs can be secreted from parasites during the early stages of amastigogenesis in vitro at pH 5.0 [8,9]. It has also been proposed that the expression profile of these proteins could change during the course of infection and that different strains could differentially express several members of this family [6]. It has also been shown that immature MASP proteins, as well as the C-terminal portion, can be contained in trypomastigote exovesicles and have immunogenic properties [10,11].

Furthermore, expression of these proteins in cell membranes is variable in the parasite population [12]. The characterization of a 52-kDa MASP secreted during a parasite’s interaction with target cells has been reported [13,14]. Likewise, it has been reported that several family members are essential during the infection process of *T. cruzi* in vitro and in vivo [15,16]. Additionally, MASPs have immunogenic properties, and some peptides derived from these proteins have been studied as candidates for vaccines against *T. cruzi* and serological diagnosis [17,18,19]. In addition, two subgroups of MASP proteins could be the most immunogenic during the course of infection, at least in a murine model [6].

The present work aimed to study the presence of MASP49 in the three developmental stages of *T. cruzi.* Furthermore, its role in the infection process, glycosylation characteristics, binding to target cells, and interaction with a receptor in mammalian cells were evaluated. The results thereof suggest that MASP49 can be considered a virulence factor for *T. cruzi.*

## 2. Materials and Methods

### 2.1. Parasites

The Mexican *T. cruzi* strains MHOM/MX/1994/Ninoa and TBAR/MX/0000/Querétaro, which belong to discrete typing unit 1 (DTU 1), were employed in this work. Genetic and biological characterization of these strains has been previously published [20,21,22,23,24]. Epimastigotes were grown at 28 °C in a liver infusion tryptose (LIT) medium with 25 mg/L hemin (Sigma, St Louis, MO, USA) supplemented with 10% heat-inactivated fetal bovine serum (FBS, Invitrogen, Waltham, MA, USA) at an initial concentration of 5 × 10^6^ parasites/mL [20]. Cell-derived trypomastigotes (CD-tryps) were obtained from infected Vero cells grown at 37 °C in a 5% CO_2_ atmosphere in Dulbecco´s minimal essential medium (DMEM, Invitrogen, Waltham, MA, USA), supplemented with 10% FBS, 100 mg/mL streptomycin, and 100 U/mL penicillin (Invitrogen, Waltham, MA, USA). Briefly, Vero cells were cultured in 75 cm^2^ Corning cell culture flasks and, after 3 days of culture, were infected with 2 × 10^6^/mL of trypomastigotes per ml of cultured cells, and each third day, the culture supernatant that contained the parasites was collected and centrifuged at 700× *g* for 10 min. The pellet that contained the parasites was cultured at 37 °C for two hours to obtain swimming CD-tryps. Then, the medium was centrifuged to 2500× *g* to obtain the parasites. As determined through microscopy, nearly 90% of the recovered parasites had trypomastigote morphologies. Amastigotes were obtained through in vitro amastigogenesis. Briefly, tissue-culture-derived CD-tryps (5 × 10^6^) were harvested as described before, transferred to 25 cm^2^-cell culture flasks with 1 mL of DMEM at pH 5, and incubated at 37 °C in a 5% CO2 atmosphere for 24 h [25]. More than 90% of the parasites presented morphologies that corresponded to the amastigote stage determined through microscopy observation.

### 2.2. Peritoneal Macrophages

Peritoneal macrophages were obtained from wild-type and C-type lectin receptor-deficient C57BL/6 mice (KO-mMGL) through administration of 1 mL of 3% thioglycolate (Sigma, St. Louis, MO, USA) to elicit peritoneal macrophage recruitment [26]. Three days later, peritoneal cells were recuperated through peritoneal washing with 10 mL of cold PBS and centrifuged at 290× *g* for 10 min at 4 °C. These cells were washed three times with PBS, resuspended in DMEM, and seeded into glass Petri dishes to obtain adherent cells. Following 5 h of incubation at 37 °C in a 5% CO_2_ atmosphere, nonadherent cells were discharged. Macrophages were detached via incubation in 5 mM of cold EDTA over an ice bed for 15 min. The cells were centrifuged as above, counted in a Neubauer chamber, and adjusted to 1.5 × 10^5^ cells/mL. They were then used for MASP49 binding assays or in vitro infection assays.

### 2.3. Rat Cardiomyocytes

Ventricular myocytes were isolated from Wistar rats using an enzymatic perfusion method [27]. Briefly, the rats were treated with heparin (1000 U/kg) (Sigma, St. Louis, MO, USA) and anesthetized with Na+ pentobarbital (50 mg/kg) (Sigma, St. Louis, MO, USA). Beating hearts were surgically removed. These hearts were placed on ice in an oxygenated Tyrode solution (NaCl 130 mM; NaH_2_PO_4_ 0.4 mM; NaHCO_3_ 5.8 mM; MgCl_2_ 0.5 mM; KCl 5.4 mM; glucose 22 mM; HEPES 25 mM) (Sigma, St. Louis, MO, USA) and transferred to the Langerdoff system. An EGTA solution (0.1 mmol/L) (Sigma, St. Louis, MO, USA) was perfused to clarify the hearts. Myocardial tissue fragments were dissected in a Petri dish that contained an enzyme solution (1 g/L of collagenase, 0.1 mmol/L of CaCl_2_, 2 g/L of bovine serum albumin) (BSA, Sigma, St. Louis, MO, USA). The single-cell suspension was centrifuged at 65× *g* for 3 min to minimize noncardiomyocyte contamination. The cell pellet was washed in a Tyrode solution supplemented with 0.5 mM of CaCl_2_ and stored in Tyrode solution supplemented with 1 mM of CaCl_2_ and 2 g/L of BSA. The cells were used immediately for MASP49 binding assays.

### 2.4. MASP Peptide and Antiserum Production

A MASP peptide was designed using CLC Protein Workbench software (https://www.selectscience.net/products/clc-free-workbench-software/?prodID=20301 (accessed on 2 October 2022)) based on several criteria used in similar studies, including selection of the most conserved motifs present in the predicted protein, prediction of peptide immunogenicity, and specificity of peptides to MASPs [12]. A peptide that corresponded to the amino-terminal region (Cys-APNKRAADSSSE) was synthesized (Zymed, San Francisco, CA, USA). A cysteine residue was added to allow conjugation to the keyhole limpet hemocyanin (KLH) carrier protein. An antiserum for this peptide was generated through two subcutaneous rabbit immunizations with 500 µg of peptide and Freund’s complete adjuvant (Sigma, St. Louis, MO, USA), followed by four more immunizations using Freund´s incomplete adjuvant (Sigma, St. Louis, MO, USA) at 15-day intervals. The anti-MASP serum was divided into aliquots and stored at −20 °C. For some experiments, antibodies were purified through affinity chromatogphy using protein G (Invitrogen, Waltham, MA, USA), following the procedure described previously [28]. The IgG purified antibodies were quantified via determination of their absorbances at 260 and 280 nm in a Ultrospec III UV/Vis Spectrophotometer (American Laboratory Trading, Lime, CT, USA) and application of the formula mg/mL = (1.55 × Abs_280_) − (0.775 × Abs_260_).

### 2.5. Total Protein Extract

Total protein extracts were obtained from epimastigotes, amastigotes, and CD-tryps via direct disruption of the parasites in a lysis buffer (7 M of urea, 2 M of thiourea, 4% CHAPS, and 10 mM of Tris) (Sigma, St. Louis, MO, USA) in a 1:3 ratio parasite weight/buffer volume in the presence of protease inhibitors (12 mM of EDTA, 1 mM of PMSF, 0.1 mM of leupeptin, 1 µM of pepstatin) (Sigma, St. Louis, MO, USA). The soluble fraction was obtained through centrifugation at 12,800× *g* for 15 min. A 2D-Quant kit (GE Healthcare, Chicago, IL, USA) was employed for protein quantification [29].

### 2.6. MASP49 Immune Detection

Western blotting was used to detect MASP49, as previously described [30]. Briefly, total protein extracts (50 μg) were loaded in preparative 12% SDS-PAGE and transferred to nitrocellulose membranes. The membranes were cut into 3 mm strips, which were incubated with 10% nonfat dry milk (Nestle, Vevey, Swiss) overnight. Next, MASP was detected using rabbit anti-MASP49 antiserum diluted 1:500 in PBS-0.1% Tween 20 (Sigma, St. Louis, MO, USA) for 2 h at room temperature. Finally, the blots were incubated with HRP antirabbit IgG (Zymed, San Francisco, CA, USA), diluted (1:6000), and visualized with 3,3-diaminobenzidine (Sigma, St. Louis, MO, USA).

### 2.7. Indirect Immunofluorescence (IFI) Assay 

The parasites were fixed in 2% paraformaldehyde/PBS (Sigma, St. Louis, MO, USA), spotted on a clean glass slide, and allowed to dry. The fixed parasites were blocked for 2 h with 2% BSA in PBS (BSA-PBS). The slides were incubated with 10 µg/µL of IgG anti-MASP49 antibodies or rabbit IgG isotypes (Biolegend, San Diego, CA, USA) overnight at 4 °C. The slides were washed three times with a 1% BSA solution and incubated with Alexa Fluor 594-conjugated donkey antirabbit IgG (Invitrogen, Waltham, MA, USA) at a 1:250 dilution for 1 h. The slides were washed, mounted, and examined in an Olympus BX51-WI DSU microscope (Olympus Life Science, Center Valley, PA, USA) using a 100 × oil-immersion objective.

### 2.8. Membrane Enriched Fraction

A membrane protein (MP) enrichment fraction was obtained as described previously, with some modifications [31]. Briefly, epimastigotes from the Qro strain at the exponential growth phase were collected in a lysis buffer (400 mM of mannitol, 10 mM of KCl, 2 mM of EDTA, 1 mM of PMSF, 10 mg/mL of leupeptin, 5 mg/mL of aprotinin, 20 mM of HEPES, pH 7.4) (Sigma, St. Louis, MO, USA). Lysis of the parasites was performed with five cycles of freezing–thawing. After differential centrifugation at 1000× *g* for 10 min for separation of nuclei, followed by centrifugation at 16,000× *g* for 30 min to eliminate mitochondria, a fraction enriched in membrane proteins was obtained through centrifugation of the supernatant at 100,000× *g* for 1 h. This fraction was stored at −20°C until used. Five μg of enriched surface proteins and total extract were separated with 12% SDS-PAGE and transferred to a nitrocellulose membrane (Amersham Biosciences, Amersham, UK). Western blotting was performed using anti-MASP49 (3 μg/mL) and HRP antirabbit IgG (Invitrogen, Waltham, MA, USA).

### 2.9. MASP49 Purification

The total protein extract from the epimastigotes of the Ninoa strain was separated based on size-exclusion chromatography in Superose 12 columns (Amersham Biosciences, Amersham, UK). First, fractions containing MASP49 were detected with Western blot, using the anti-MASP antibody. Then, these fractions were separated with 8% acrylamide gel electrophoresis in a tricine discontinuous system (Bio-Rad, Hercules, CA, USA) [32]. Finally, the MASP49 protein was excised from the gel and eluted in PBS overnight at 4 °C.

### 2.10. Phosphatidylinositol-Specific Phospholipase C (PLC) Treatment

CD-tryps (100 × 10^6^) from the Ninoa strain were washed three times with PBS and resuspended in 500 µL of DMEM without FBS. The parasites were incubated for 2 h at 37 °C with or without an addition of 4 U of PLC from *Bacillus cereus* (Sigma, USA) [33]. The supernatants were collected and centrifuged at 12,800× *g* for 15 min and filtered through a 0.45 µm membrane (Millipore, Burlington, MA, USA). Proteins from supernatants were quantified using Bio-Rad’s DC protein assay (Bio-Rad, Hercules, CA, USA), and 10 mg were loaded into 12% SDS-PAGE. Western blotting was performed as described above using anti-MASP49 serum (diluted 1:500) or anti-trans-sialidase antibodies (α-TS) (diluted 1:100): a gift from Dr. Sergio Schekman, Scola Paulista do Medicina, Brazil.

### 2.11. In Vitro Inhibition Infection Assay

The ability of purified IgG anti-MASP49 to inhibit CD-tryp invasion of Vero cells was evaluated as reported previously [34]. Briefly, Vero cells were seeded in 21-well hydrophobic printed slides (Electron Microscopy Sciences, Hatfield, PA, USA), at 3 × 10^3^ cells/well and in 20 μL of DMEM. These cells were incubated for 2 h to allow adherence, and 3 × 10^4^ CD-tryps of the Ninoa strain, which had been previously incubated with DMEM, increasing concentrations of IgG anti-MASP49, or rabbit IgG control isotypes for 2 h at 4 °C, were added. After 1 h of incubation, the medium that contained the noninternalized parasites was removed, and the cells were washed three times with PBS and fixed with methanol (Sigma, St. Louis, MO, USA). The cells were then stained with May–Grünwald–Giemsa staining (Sigma, St. Louis, MO, USA), and the percentage of infected cells and the number of internalized CD-tryps per 100 cells were determined with microscopy. All assays were performed in triplicate. The results thereof are presented as the medians +/− standard deviation from 2 independent assays. Analysis via one-way ANOVA with a Tukey multiple comparison test was performed using GraphPad Prism version 5 for Windows. The same assay was performed using both wild-type and KO-mMGL murine macrophages, with trypomastigotes incubated with the highest concentration of anti-MASP antibodies. Likewise, macrophages activated with 50 ng/mL of murine recombinant IL-4 (mrIL-4, R&D systems, Minneapolis, MN, USA) for 48 h were also used.

### 2.12. Binding Assay

Vero cells, murine macrophages, and rat cardiomyocytes were obtained as described above and fixed with 2.5% glutaraldehyde (Sigma, St. Louis, MO, USA). The binding assay was performed as previously described [35]. Briefly, 200 µg of total protein extract from the Ninoa and Qro CD-tryps was incubated with 1 × 10^6^ fixed cells for 18 h at 4 °C. Unbound material was removed with centrifugation, and cells with bound parasite material were washed three times with a TDSET buffer (10 mm of Tris-HCl pH 7, 10 nM of EDTA, 0.2% DOC, 0.1% SDS, and 1.0% Triton X-100) (Sigma, St. Louis, MO, USA). Proteins bound to host-cell surfaces were eluted with a sample buffer (50 mM of Tris-Cl, 2% SDS, 0.05% bromophenol blue, 10% glycerol, 100 mM of b-mercaptoethanol) (Bio-Rad, Hercules, CA, USA), separated with 12% SDS-PAGE, and transferred to nitrocellulose membranes. MASP49 was detected with Western blotting using IgG anti-MASP antibodies.

### 2.13. Glycoprotein Detection

Glycoproteins were detected using the DIG Glycan Differentiation Kit (Roche, Basel, Switzerland) following the manufacturer’s instructions. Briefly, 200 μg of total protein extracts of epimastigotes from the Ninoa strain were loaded into 8% SDS-PAGE and transferred to nitrocellulose membranes, as described in the MASP49 immune-detection section, and probed against five digoxigenin-labeled lectins: GNA (Galanthus nivalis agglutinin), SNA (Sambucus nigra agglutinin), MAA (Maackia amurensis agglutinin), PNA (Peanut agglutinin), and DSA (Datura stramonium agglutinin) (Sigma, St. Louis, MO, USA). A similar assay was performed using previously purified MASP49 protein.

### 2.14. MASP Binding Inhibition via Carbohydrates

Peritoneal macrophages were obtained from CD-1 mice and lysed in a lysis buffer (50 mM of Tris, 150 mM of NaCl, 0.5% Triton X-100, 1 mM of CaCl_2_, and 1 mM of MgCl_2_) (Sigma, St. Louis, MO, USA) with protease inhibitors, then centrifuged at 10,000× *g* for 3 min at 4 °C. ELISA plates were coated with 50 μg/mL protein extract, incubated for 2 h, and washed with a blocking buffer (1% BSA, 154 mM of NaCl, and 0.05% Tween 20). The plates were then blocked overnight at 4 °C with the buffer described above. The plates were then incubated for 2 h in the presence or absence of different concentrations (10, 20, and 80 mM) of galactose, mannose, or glucose (Sigma, St. Louis, MO, USA). After incubation, the plates were washed as described above. These plates were incubated for 2 h with 5 μg of extract from CD-tryps, washed before addition of rabbit anti-MASP (10 μg/µL), incubated for 2 h, and washed as described above. Rat anti-F4/80 (Caltag, Burlingame, CA, USA, 1:200) or rat anti-mMGL (Santa Cruz, Dallas, TX, USA, 1:50) was used directly after the macrophage protein extract as a control. Secondary antibodies were added (anti rat-HRP or anti rabbit-HRP, 1:1000) (Invitrogen, Waltham, MA, USA) for 2 h. Finally, the enzymatic reaction was developed with O-phenylenediamine (Sigma, St. Louis, MO, USA). Absorbance values were determined with an ELISA reader (Bio-Rad, Hercules, CA, USA, Model 3550) at 490 nm. Two independent experiments were carried out in duplicate. Significant differences were calculated with a two-tailed Student’s t-test using online GraphPad QuikCalc Software (http://www.graphpad.com/quickcalcs/ttest1.cfm (accessed on 2 October 2022)).

### 2.15. The Colocalization of MASP49 and mMGL

Peritoneal macrophages (3 × 10^4^/ well) from C57BL/6 mice were placed in 12-well slides and grown overnight at 37 °C. First, nonadherent cells were removed, and 50 ng/mL mrIL-4 was added. Next, the cells were incubated at 37 °C for 48 h. After fixation with 2% paraformaldehyde–PBS, the cells were washed with PBS three times and blocked with PBS that contained 10% FCS (Invitrogen, Waltham, MA, USA) (FCS-PBS) for 1 h at room temperature. Next, they were incubated with 5 mg of total protein extract from the CD-tryps in FCS-PBS for 1 h at 37 °C, and the cells were then washed in PBS and incubated overnight at 4 °C with IgG anti-MASP49 antibodies (10 µg/mL) and rat anti-mMGL (10 µg/mL) (Santa Cruz, Dallas, TX, USA) diluted in 0.5% BSA-PBS. Finally, Alexa Fluor 594-conjugated donkey antirat or Alexa Fluor 488-conjugated donkey antirabbit (Invitrogen, Waltham, MA, USA), at a 1:250 dilution, was added. The slides were examined under a confocal LSM 5 Pascal microscope (Carl Zeiss, Oberkochen, Germany) [36].

### 2.16. Animal Ethical Management

All experiments that involved animals followed the ethical guidelines of the ethical code of Instituto de Investigaciones Biomédicas, Universidad Nacional Autónoma de México (https://www.biomedicas.unam.mx/wp-content/pdf/intranet/reglamentos/codigo-etico-iibo.pdf?x30807 (accessed on 2 October 2022)). Institutional Commission for the Care and Use of Laboratory Animals; approval code: ID 150; approval date: 30 of July 2021.

## 3. Results

### 3.1. MASP49 Is Expressed in All Stages of T. cruzi

**Figure 1:**
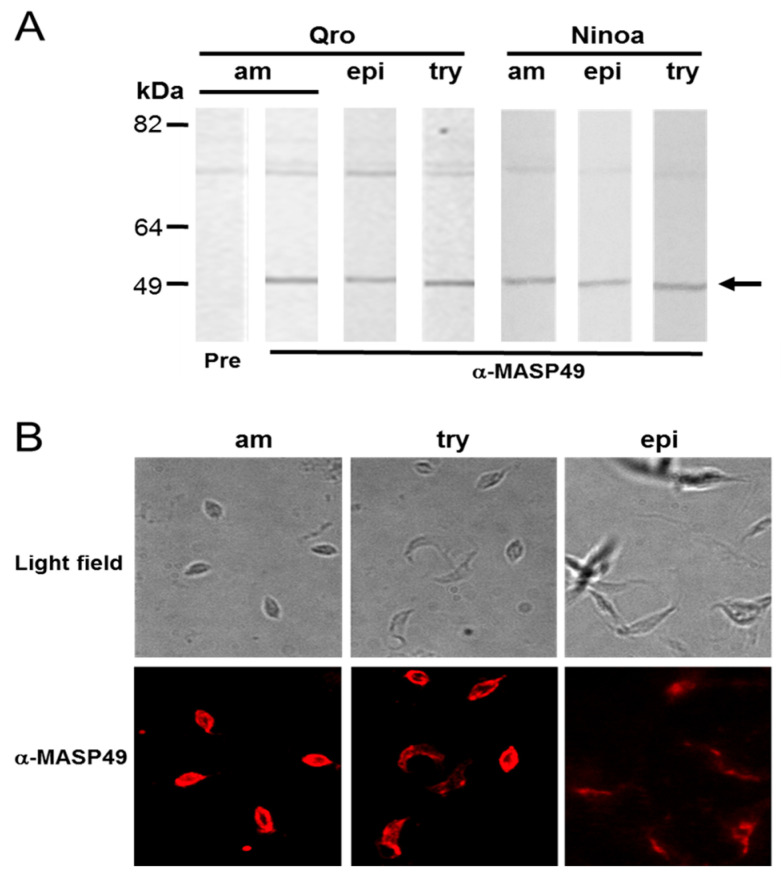
Expression and cellular localization of MASP49 in *T. cruzi* stages. (**A**) Western blot analysis was performed using rabbit preimmune serum (Pre) or rabbit serum α-MASP49 (α-MASP49) against total protein extracts from amastigotes (am), epimastigotes (epi), and CD-tryps (try) of the Querétaro (Qro) and Ninoa strains, as described in Materials and Methods. MASP49 recognition is indicated with an arrow. (**B**) The surface localization of MASP49 was evaluated through IFI assay in all stages of the Qro *T. cruzi* strain, using affinity-purified α-MASP49 antibodies and Alexa conjugated donkey antirabbit IgG, as described in Materials and Methods. Light microscopy (upper panel) and confocal microscopy (lower panel) are shown.

Total protein extracts from the Mexican Querétaro (Qro) and Ninoa TcI strains were obtained from amastigotes, epimastigotes, and cell-derived trypomastigotes (CD-tryps). These were used to evaluate MASP expression at different developmental stages of *T. cruzi*. Rabbit serum produced against an amino-terminal peptide (α-MASP49) recognized a 49-kDa protein (MASP49) in the extracts of the three stages of both strains (Figure 1A). There was a nonspecific band, of approximately 70 kDa, that was not considered for analysis because the preimmune sera also recognized this band (Figure 1A). The 49-kDa protein was also expressed in epimastigotes from the South American Silvio TcI strain; in this case, other weak signals were observed (data not shown). Additionally, the localization of MASP49 on the Qro parasite surface was determined through indirect immunofluorescence (IFI). Differential MASP49 distribution was observed on the cell surface of the three stages studied in this work. In amastigotes, this distribution was continuous and abundant. In contrast, MASP49 on CD-tryps parasites had a differential distribution; in some parasites, it was localized in patches on the membrane. Additionally, the epimastigotes had the lowest presence of this protein on the surface (Figure 1B).

### 3.2. MASP49 Is GPI-Anchored to the Membrane of the Parasite

**Figure 2:**
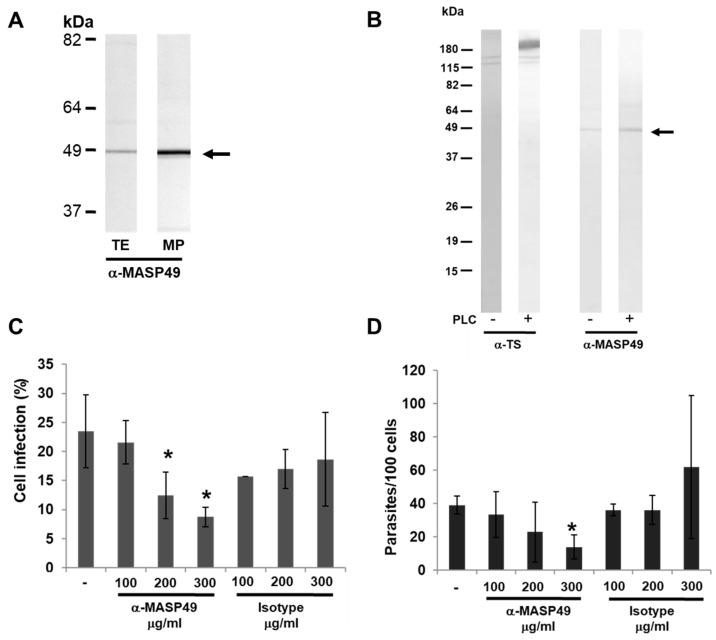
GPI anchor of MASP49 and its role in *T. cruzi* cell invasion. (**A**) MASP49 from the Ninoa epimastigote total extract (TE) and from MP-enriched fraction obtained via differential centrifugation was detected with Western blotting using rabbit serum α-MASP49 (arrow). (**B**) Supernatants from Ninoa CD-tryps were obtained after parasite incubation with or without 4 U of PLC for 2 h at 37 °C and analyzed through Western blotting using rabbit serum α-MASP49 or mouse monoclonal anti-trans-sialidase antibodies (α-TS). MASP49 was detected in the supernatant (arrow). (**C**) Percentages of cell infection after pretreatment of Ninoa CD-tryps with increasing concentrations of α-MASP49 antibodies (µg/mL). (**D**) Intracellular parasites per 100 cells after pretreatment of Ninoa CD-tryps with increasing concentrations of α-MASP49 antibodies. As controls, the isotype antibody concentration (µg/mL) and parasites without antibody incubation (−) are included. The values are the means ± SD of three independent experiments performed in triplicate. * *p* < 0.05.

The presence of MASP49 was observed in a MP-enriched fraction obtained from the Ninoa-strain *T. cruzi* epimastigotes (Figure 2A). This fraction contained internal and membrane-associated surface proteins. The presence of a glycosylphosphatidylinositol (GPI) anchor on the MASP49 from the Ninoa CD-tryps was evaluated through treatment with phospholipase C (PLC). Identification of trans-sialidase (TS), which is known as a GPI-anchored protein, was used as a positive control. A slight increase in MASP49 was observed in the medium after incubation with PLC (Figure 2B). In this strain, part of the MASP49 was released to the medium without incubation with PLC.

### 3.3. MASP49 in Mammalian Infection

Infection assays were performed to establish whether MASP49 has a role in infection of mammalian cells. Preincubation of the Ninoa CD-tryps with the α-MASP49 antibody reduced the entry of parasites into Vero cells in a dose-dependent manner, and this decrease was significant when 200 and 300 µg/mL of the antibodies were used (*p* < 0.05) (Figure 2C). The reduction in infection was specific, since the rabbit IgG isotype, used as a control, did not significantly affect parasite entry. Additionally, we evaluated the number of intracellular parasites that showed a significant decrease in number of amastigotes when these parasites were treated with 300 µg/mL of α-MASP49 antibodies (*p* < 0.05) (Figure 2D).

### 3.4. MASP49 Binds to Different Host Cells

**Figure 3:**
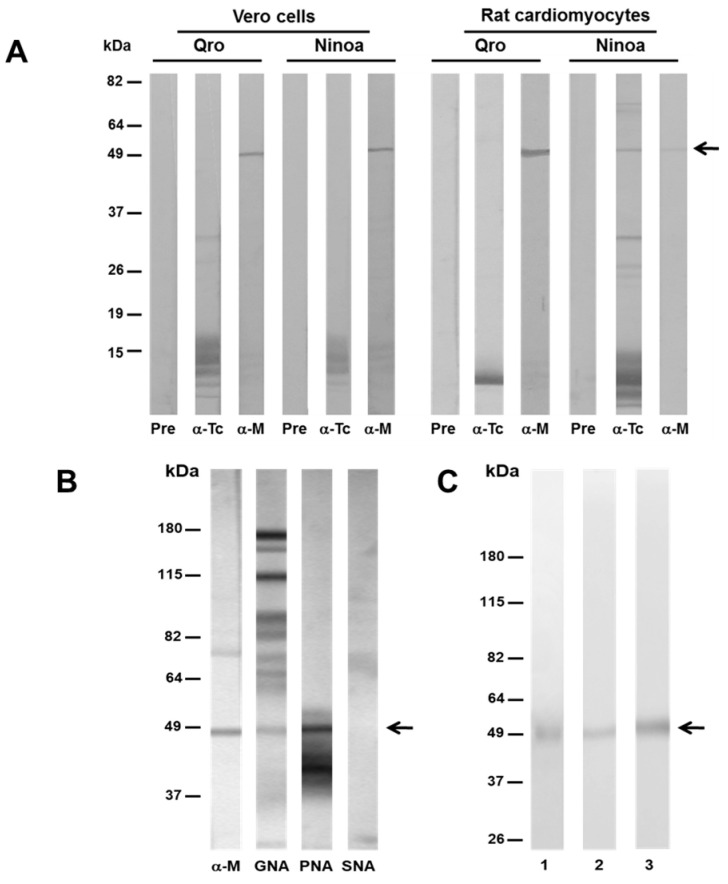
Binding assay and MASP glycosylation. (**A**) Fixed Vero cells or rat cardiomyocytes from primary cultures were incubated with CD-trys protein extract from Qro and Ninoa strains, as described in Materials and Methods. Eluted binding proteins were detected with α-*T. cruzi* total protein antibodies (α-Tc) or α-MASP49 antibodies (α-M) through Western blotting. Preimmune serum (Pre) was used as a control. (**B**) The Ninoa-epimastigote total protein extract was blotted on nitrocellulose membranes and probed against anti-MASP49 antibodies (α-M), GNA (Galanthus nivalis agglutinin), PNA (Peanut agglutinin), and SNA (Sambucus nigra agglutinin), as described in Materials and Methods. (**C**) MASP49, purified with size-exclusion chromatography and glycine gels, was visualized with Coomassie blue (1) and detected using anti-MASP49 antibodies (2) or PNA lectin through Western blotting (3). Arrow: 49 kDa protein detection.

A binding assay was performed to determine whether MASP49 binds to surface proteins of Vero cells and primary-culture rat cardiomyocytes, as described in Materials and Methods. In the Ninoa- and Qro-eluted proteins from the Vero cells, a band of 49 kDa, with similar intensity, was recognized by α-MASP49 antibodies (Figure 3A). In the case of the rat cardiomyocytes, the intensity of the recognized 49 kDa was more evident when the extract of the Qro strain was used. As a control, an antibody against *T. cruzi* proteins was used.

### 3.5. MASP49 Is Glycosylated with Galactose (1-3) N-Acetylgalactosamine

We used the DIG Glycan Differentiation Kit to assess whether MASP49 is a glycoprotein. Only PNA and GNA lectins that recognized galactose (1-3), N-acetylgalactosamine (GalNac), and mannose, respectively, gave positive signals with the Ninoa proteins (Figure 3B). Among them, a 49-kDa protein with the same molecular weight as the protein that was recognized by the α-MASP49 antibody was identified. Furthermore, the MASP49 was purified using chromatography and protein elution from a Tricine acrylamide gel system. The different fractions obtained from the purification process were evaluated through the SDS-PAGE and Western blotting using the α-MASP49 antibodies. In some of these fractions, only a 49 kDa protein was observed (Figure 3C1). The same protein was recognized by the α-MASP49 antibodies (Figure 3C2). Furthermore, only PNA recognized the purified MASP49, confirming the presence of GalNac in the epimastigote protein (Figure 3C3). No recognition with SNA was observed using the purified MASP49, confirming our previous results with the total epimastigote extract.

### 3.6. MASP49 Binds to mMGL, and Galactose Reduces This Binding

**Figure 4:**
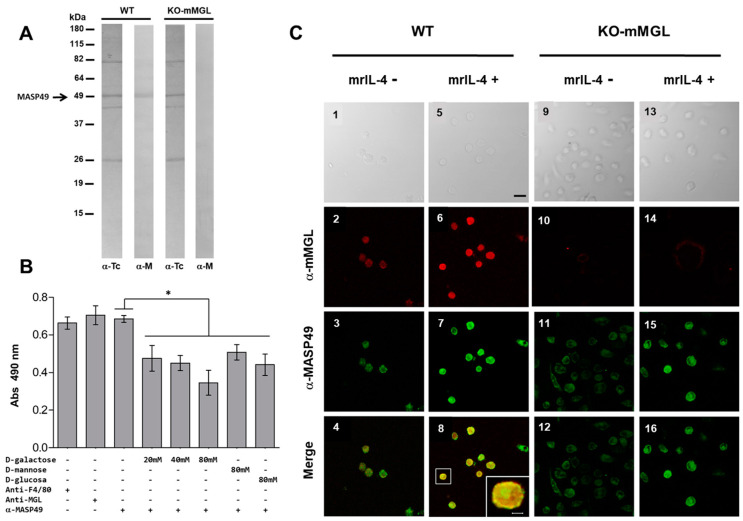
MASP49 binding to mMGL. (**A**) A binding assay was performed using fixed peritoneal macrophages from WT or KO-mMGL C57BL/6 mice and the total Qro CD-tryps protein extract. The binding proteins were detected with rabbit anti-*T. cruzi* serum (α-Tc) or anti-MASP49 antibodies (αM), as described in Materials and Methods. (**B**). ELISA was performed using the WT macrophage extract in the presence or absence of several carbohydrates and the extract from CD-tryps. Anti-MASP49, anti-F4/80, or anti-mMGL antibodies were used. Values are presented as median +/− standard deviation (* *p* < 0.05). (**C**) MASP49 binding to mMGL was evaluated with confocal microscopy using peritoneal macrophages from WT or KO-mMLG C57BL/6 mice in the presence and absence of mrIL-4. MASP49 (green) and mMGL (red) on the surface were revealed through Alexa conjugates. A merge of both images was created. Scale bar represents 50 µm (black) and 10 µm (white).

Due to the confirmed presence of GalNac, the MASP49 could be recognized by carbohydrate receptors in mammalian cells. In addition, the murine receptor for C-type lectins (mMGL) is known to have an affinity for GalNac. Therefore, peritoneal macrophages from wild-type C57BL6 mice (WT) and mMGL knockout mice (KO-mMGL), which lacked the C-type lectin receptor, were used in a binding assay, as previously described. The MASP49 bound to the surface of the WT macrophages but not the KO-mMGL macrophages. As a control, an antibody directed against total *T. cruzi* proteins was used (Figure 4A). To establish the importance of the GalNac present in MASP49 during binding to mMGL, an inhibition assay was performed using several carbohydrates, including GalNac. The presence of galactose reduced the binding of MASP49 to mMGL in a dose-dependent manner (Figure 4B). Interestingly, mannose and glucose also reduced MASP49–mMGL binding but were less efficient, since higher carbohydrate concentrations produced less binding inhibition.

A colocalization assay was performed to test the binding of MASP49 to mMGL on the surfaces of macrophages. Under basal conditions, MASP49 and the mMGL receptor colocalized (Figure 4C 1, 2, 3, 4). Additionally, since the expression of the mMGL receptor increased after addition of mrIL-4, this cytokine was added to macrophages from wild-type mice. When mrIL-4 was added, the mMGL receptor on the surface of the WT macrophages increased. This correlated with more binding of MASP49 detected via specific antibodies (Figure 4C 5, 6, 7, 8). The colocalization of both lectin and MASP49 was confirmed when these signals were merged (Figure 4C8 and the amplification in the box). Colocalization was not observed in a similar experiment with macrophages from mMGL knockout mice in the absence (Figure 4C 9, 10, 11, 12) or presence of mrIL-4 (Figure 4C 13, 14, 15, 16).

### 3.7. The Absence of mMGL Reduces Peritoneal Macrophage Infection

**Figure 5:**
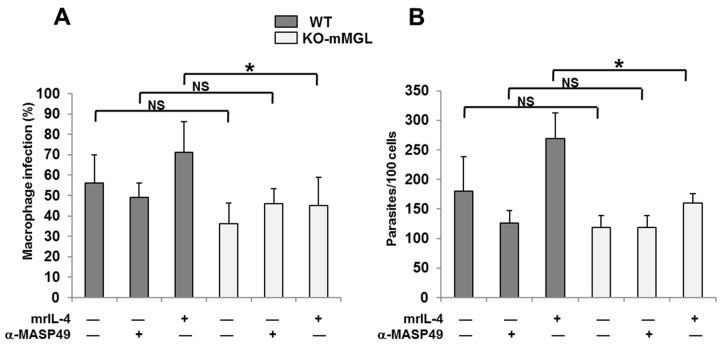
Infection of WT and KO-mMGL C57BL/6 macrophages. An in vitro infection assay was performed, as reported in Materials and Methods, using parasites pretreated or not with 300 µg/mL of α-MASP49 antibodies. In some experiments, mrIL-4 was added. (**A**) The percentage of infected cells and (**B**) intracellular parasites per 100 cells were counted. The values are shown as the means ± SD of three independent experiments performed in triplicate (* *p* < 0.05); not significant (NS).

In vitro infection assays were performed using macrophages from WT and KO-mMGL C57BL/6 mice in the presence or absence of mrIL-4. A reduction in the number of infected cells in WT macrophages was observed when parasites were preincubated with α-MASP49 antibodies (Figure 5A). However, compared with the control condition, it was not statistically significant. When the mrIL-4 was added to the WT cells, the average number of infected cells increased, but this increase was not statistically significant. On the other hand, the infection percentage of KO-mMGL macrophages was lower compared with that of the WT, but it was not statistically significant. As expected, the mrIL-4 increased the number of WT macrophages infected but had no effect on the KO-mMGL cells. Similar results were observed in evaluation of the number of intracellular parasites in WT and KO-mMGL macrophages infected with *T. cruzi* (Figure 5B).

## 4. Discussion

Trypomastigote surface proteins have been widely studied because of their importance in parasite biology. These proteins appear to play a role in the parasite’s defense against the host’s immune system and its binding and entry into host cells [3]. Several proteins have been proposed to be involved in the binding–invasion process, but which surface proteins interact specifically with a host-cell receptor is poorly known. The interaction in these regions seems to be mediated in part by GPI-anchored surface proteins [37].

Almost 25% of the diploid parasites genome codes for surface proteins, including widely studied families such as trans-sialidases and mucins [38,39]. With the publication of this parasite’s genome in 2005, two families of surface proteins were described for the first time: gp63, which is homologous to the gene in *Leishmania sp.* parasites, and a second family of proteins, called mucin-associated surface proteins (MASP). The MASP family encodes genes that correspond to nearly 6% of the complete *T. cruzi* genome [4].

In this work, we produced an antibody against a synthetic 13-amino acid peptide that corresponded to a conserved amino-terminal region of a MASP. Using this antibody, the presence of MASP49 was detected in the protein extracts of the three developmental stages in two Mexican strains. It was localized on the parasite surface, but with differential distribution, with amastigotes showing the stronger presence, CD-tryps showing surface patches, and epimastigotes having a lower signal. This result contrasts with a previously published report that described the expression of a different 45 kDa MASP protein found only in trypomastigotes but not in epimastigotes [12]. These data suggest that there are at least two different MASPs, albeit with near molecular weights to one another. These differences may be due to differential expressions in several DTUs and to the polymorphism in this protein family, as previously proposed [12].

Furthermore, the MASP49 described in our study was associated with membrane-enriched fractions in epimastigotes and partially anchored to the cell membrane with GPI, showing that MASP49 is located on the parasite surface, as was reported for other MASPs [12]. Additionally, incubation of CD-tryp with anti-MASP49 antibodies reduced the percentage of Vero-infected cells and the number of amastigotes, showing that MASP49 also participates in the binding–invasion process of *T. cruzi* on mammalian cells, as has also been reported for other members of this family [13].

Although some immunogenic and biological properties of members of the MASP family have been evaluated, little is known about the properties that allow them to interact with mammalian cells and what molecules can act as receptors. Our results show that MASP49 can bind to molecules on the surfaces of epithelial cells and, importantly, on cardiomyocytes. The latter are the primary target cells of the *T. cruzi* parasite in mammals, including humans.

Additionally, our data show the presence of GalNac in MASP49. Previous works have reported that epimastigotes and trypomastigotes of several strains of *T. cruzi* express galactose and N-acetyl-D-galactosamine residues on their surface [40,41]. Furthermore, it has been reported that some surface proteins of macrophages participate in recognition of β-D-galactose and N-acetyl-D-galactosamine residues present in trypomastigote surface proteins, which facilitates entry into cells [42,43].

The results that showed that MASP49 does not bind to surface proteins of peritoneal macrophages that lack mMGL receptors suggest that MASP49 can bind to this receptor through GalNac directly or in combination with some other protein; this last aspect must be investigated further. The colocalization study showed that the mMGL receptor is one of the possible receptors for MASP49 on the surface of macrophages, as has been demonstrated for other pathogens [36]. Furthermore, the previously reported increased expression of this receptor via mrIL-4 [44] also increased the binding of MASP49, giving additional funding to the role of the mMGL receptor as one of the sites for MASP49 binding, alone or in association with other proteins. 

Competition experiments that used several carbohydrates have demonstrated that this binding is diminished preferentially with D-galactose, further supporting the role of mMGL as one of the possible receptors for MASP49. Less reduction in binding of MASP49 to macrophages was observed with mannose and glucose, indicating that MASP49 may also bind to other receptors. Previous work has shown that some MASPs could be recognized by the Con A lectin, suggesting that some members of this family might be glycosylated with mannose and glucose [7].

Finally, the involvement of MASP49 in the infection process was demonstrated when the IgG anti-MASP49 significantly blocked parasite entry and reduced the number of intracellular amastigotes in the Vero cells. When macrophages from C57BL/6 mice were used for similar experiments, cells from WT mice showed higher percentages of infected macrophages than did Vero cells. Then, the cells from the WT mice were preincubated with mrIL-4 (a macrophage activator), and there was an increase in infection and intracellular amastigotes. However, the reduction after incubation with anti-MASP49 was negligible, as was the number of parasites in the cells. When these experiments were performed with macrophages from C57BL/6 KO-mMGL mice, a reduction in the percentage of infection and the number of parasites per cell was observed compared with those of the WT mice.

In general, these results indicate a role for the lectin receptor in the infection phenomenon and showed that the *T. cruzi* parasite has different pathways to enter each type of cell. Parasite entry into different cell types shows specific invasive mechanisms into phagocytic versus nonphagocytic cells, as has been reported before [26]. These diverse mechanisms can cause the differences observed in our work between infection and inhibition of infection in Vero cells and macrophages.

The results obtained in this work provide more information about a possible interaction between MASPs and host cells. Knowing that MASP49 is glycosylated with GalNac opens the possibility of looking for more receptors for this carbohydrate in cells of various tissues to better understand the role of this protein during the infection process. In cardiomyocytes, the molecule involved in binding to MASP could be a receptor for mannose, galactose, or both, since it has previously been shown that infecting cardiomyocytes with *T. cruzi* in the presence of mannose or galactose reduces parasite entry [45].

The results of this work, together with those published by other groups, show that MASPs could be considered virulence factors, since they play an important role in the entry of the *T. cruzi* parasite into a host cell. In addition, the importance of the mMGL receptor in the host–parasite relationship should also be considered [46,47]. Thus, it is necessary to extend this initial characterization of MASP49 and of other MASPs to understand their participation in the infection process: in particular, their binding capacity to the mMGL receptor alone or while interacting with other parasite proteins.

## 5. Conclusions

This work showed that MASP49 binds to peritoneal macrophages and rat cardiomyocytes, undergoes glycosylation via galactose N-acetylgalactosamine, and can attach to the macrophage murine C-type lectin receptor (mMGL) in mammalian cells. Importantly, its inhibition with a specific antibody reduces infection of Vero cells. All of these results indicate a possible role of this protein as a virulent factor. Additional studies must be carried out to corroborate the possible role of this protein related to receptors on mammal cells.

## Data Availability

Raw data and figures are available upon request to the corresponding authors.
